# Increasing resistance to multiple anthelmintic classes in gastrointestinal nematodes on sheep farms in southwest England

**DOI:** 10.1002/vetr.1531

**Published:** 2022-03-26

**Authors:** Katie Bull, Mike J. Glover, Hannah Rose Vineer, Eric R. Morgan

**Affiliations:** ^1^ Bristol Veterinary School University of Bristol Bristol UK; ^2^ Torch Farm & Equine Vets South Molton Devon UK; ^3^ School of Biological Sciences Queen's University Belfast Belfast UK; ^4^ Present address: Institute of Infection, Veterinary and Ecological Sciences University of Liverpool Neston UK

**Keywords:** anthelmintic resistance, faecal egg count reduction tests, *Haemonchus contortus*, sheep

## Abstract

**Background:**

Anthelmintic resistance (AR) in gastrointestinal nematodes (GIN) is increasing globally, and farmers are encouraged to adopt sustainable control measures. *Haemonchus contortus* is increasingly reported in the UK, potentially complicating effective GIN control.

**Methods:**

Faecal egg count reduction tests (FECRT) were conducted on 13 farms in north Devon, England, UK in 2016. Relative abundance of *H. contortus* was quantified using peanut agglutinin staining and used to estimate faecal egg count reduction percentages (FECR%) using the eggCounts R package.

**Results:**

On average, farms had GIN resistance to three anthelmintic classes. No farms had susceptibility to all anthelmintics tested. AR was more prevalent in 2016 than on the same farms in 2013. *H. contortus* was present on 85% of the farms tested and comprised on average 6% (0%–52%) of GIN eggs before treatment. Resistance or suspected resistance to all anthelmintics tested was observed in this species on different farms.

**Conclusion:**

The results demonstrate diversity of AR profiles on farms, apparent progression of AR within a 3‐year period, and challenges detecting AR in mixed‐species infections. Where possible, interpretation of mixed‐species FECRT should take into account the relative abundance of species pre‐ and post‐treatment to identify pragmatic treatment options targeting individual genera.

## INTRODUCTION

Reduced anthelmintic efficacy against gastrointestinal nematodes (GIN) in sheep has been reported worldwide, and the presence of heritable resistance confirmed for all widely available anthelmintic classes.^1^, ^2^, ^3^, ^4^, ^5^, ^6^, ^7^ As a result, farmers and veterinarians are now encouraged to adopt parasite control practices that reduce anthelmintic usage, in order to slow the development of resistance.^8^, ^9^, ^10^ Understanding which anthelmintics are effective on an ovine holding is key to successfully implementing sustainable nematode control strategies. Among helminths of small ruminants, *Haemonchus contortus* is a particularly pathogenic blood‐feeding GIN with global distribution.^11^ Infection with *H. contortus* can cause severe anaemia leading to condition loss, reduced productivity and eventually death.^12^, ^13^ Helminth infections, including *H. contortus*, are responsible for huge economic loss, estimated most recently for the European ruminant livestock industry at €1.8 billion per year.^14^ Of this, around €38 million is attributed to anthelmintic resistance (AR), through the costs of wasted (ineffective) treatments and production loss in animals treated with ineffective anthelmintics.^14^ This figure is undoubtedly rising as AR becomes more common.^7^

The presence of mixed infections including *H. contortus*, and the significantly higher fecundity of *H. contortus* than other co‐infecting species,^15^ also challenges the interpretation and practical implications of faecal egg count reduction tests (FECRTs) to detect AR. Typically, the species composition in FECRTs is reported as species present before and after treatment,^16^ without considering the percentage reduction in faecal egg count (FEC) attributed to each species present. However, a more detailed understanding of the anthelmintic‐resistant species provides veterinarians and farmers with a wider range of options for control. For example, on a farm where resistance to ivermectin is present in *H. contortus* but not *Teladorsagia circumcincta*, it may be possible to continue to use ivermectin to control *T. circumcincta*, targeting *H. contortus* with other, narrow‐spectrum anthelmintics such as closantel.

Many countries in the northern hemisphere are at the edge of the geographic range of climatic suitability for *H. contortus* due to limited opportunities for overwinter survival on pasture.^17^, ^18^ In these regions, *H. contortus* is able to persist by winter arrest in mature sheep,^13^, ^19^ but its presence on a farm is generally only confirmed following outbreaks of clinical haemonchosis. As cases of haemonchosis increase in this range‐edge population^20^, ^21^ against the backdrop of a high prevalence of AR, early detection of *H. contortus*, and anthelmintic‐resistant *H. contortus* in particular, is increasingly important to prevent damaging outbreaks.

A survey carrying out FECRTs on 27 farms in the southwest England in September 2013 identified widespread AR in the sample population.^22^ However, the species involved were not identified. Therefore, in the present survey, FECRTs were repeated on these farms in order to evaluate any changes in AR status between 2013 and 2016, assess the prevalence of *H. contortus* in the sample population, and, finally, differentiate between resistant *H. contortus* and other resistant nematodes to inform sustainable management of GINs on affected farms. The performance of standard methods for the detection of AR in mixed infections is evaluated and recommendations made for the future detection of AR in range‐edge populations where the target species (*H. contortus*) is not dominant and forms a variable proportion of mixed infections.

## METHODS

### Faecal egg count reduction tests

FECRTs were conducted on 13 farms in north Devon, England, UK between August and October 2016. The farms were all commercially run, with lambs born in spring and sold for meat at the end of their first grazing season. Lambs had continuous access to pasture before and after weaning, and normal parasite management included repeated treatments with anthelmintics during the grazing season. All farms had taken part in a previous FECRT survey in September 2013.^22^ Composite (pooled sample) FECs for each flock were conducted by a veterinarian at intervals between July and October 2016 using a modified McMasters method,^23^ with a detection limit of 50 eggs per gram (EPG) of faeces. Flocks with egg counts above 300 EPG were offered a FECRT as part of their ongoing veterinary care. A higher egg count threshold than recommended in the World Association for the Advancement of Veterinary Parasitology (WAAVP) guidelines^24^ was chosen due to the potential presence of *H. contortus*, taking into account the high rate of egg production by this species compared with other locally dominant species such as *T. circumcincta*.^15^ No lambs were treated with any anthelmintic within 6 weeks prior to sampling for this study. Lambs used in the study were all approximately 6 months of age, having been born in the spring of the year sampled.

Each flock was visited by veterinarians and trained technicians on Day 0, 7 and 14. On Day 0 (the day of treatment), the farm's weigh crate was calibrated using a container filled with 25 kg of sand. Individual faecal samples were taken per rectum by the veterinarian with a clean, gloved, lubricated finger and placed in individual plastic sample bags, which were then sealed. Lambs were allowed to enter the race freely in no particular order and were assigned in turn to the four treatment groups. Each treatment group was sprayed with a different colour for each anthelmintic class tested: benzimidazole (BZ; Endospec 2.5% oral drench for sheep, Bimeda) = Blue group, levamisole (LV; Levacide 3% oral drench for sheep, Norbrook) = Yellow group, ivermectin (IVM; Noramectin 0.08% oral solution for sheep, Norbrook) = Red group and moxidectin (MOX; Cydectin 1% oral drench for sheep, Zoetis) = Purple group. Lambs were then weighed to the nearest 0.5 kg and drenched over the back of the tongue using a syringe, at the manufacturer's recommended dose as determined by each lamb's individual weight (rounded up to the nearest 0.2 ml). Twenty lambs were assigned to each treatment group, except on farms where fewer lambs were available. On those farms, 20 lambs were assigned to the IVM and MOX treatment groups and a minimum of 12 lambs were assigned to the BZ and LV treatment groups. IVM and MOX groups were prioritized because resistance to BZ and LV was already known to be highly prevalent on the farms sampled^22^ and resistance to IVM and MOX was of greater interest for the benefit of the veterinary care of the flocks. One farm did not have enough animals suitable for sampling all four treatment groups so the BZ group was not included due to existing knowledge of confirmed resistance to this anthelmintic class. Faecal samples were transported directly to the University of Bristol Veterinary Parasitology laboratory for analysis.

On Day 7 (LV treatment group), or on Day 14 (BZ, IVM and MOX treatment groups), faecal samples were taken from the marked lambs as described above and either transported by next day delivery post or taken directly to the laboratory for analysis. Samples from LV‐treated lambs were collected after 7 days due to a shorter egg suppression period than for other anthelmintic groups.^24^

Due to the potentially high within‐flock prevalence of *H. contortus* in the region, a history of clinical haemonchosis on many of the participating farms, the high egg counts on some farms prior to testing, limited numbers of sheep on some farms, and the fact that the survey was carried out as part of the routine veterinary care of these flocks and not as part of research programme, the risk of disease in untreated animals was determined to be too high to allow safe inclusion of an untreated control group.

### Sample storage

On arrival at the laboratory, samples were stored at 8°C to prevent development of *H. contortus* eggs at higher temperatures and excessive mortality at 4°C.^25^ Samples were stored for a maximum of 7 days prior to processing to avoid egg deterioration.^26^

### Faecal egg counts

Due to resource constraints, individual FEC could not be conducted on all lambs sampled. The primary focus of the study was on IVM and MOX groups, given increasing concern over the efficacy of those anthelmintics since the previous study, and its serious implications for farms already known or suspected to have resistance to BZ and LV groups. Resistance to BZ and LV was already known to be present on many of the study farms,^22^ and is widespread in the UK,^7^, ^16^ and its confirmation here consequently a lower priority than for IVM and MOX. Therefore, composite (= pooled) FECs were carried out on the samples from the BZ and LV treatment groups,^27^ and individual FEC on samples in the IVM and MOX treatment groups.

Egg counts for the FECRT were conducted using a mini‐FLOTAC method sensitive to 5 EPG,^28^ with modifications used to ensure an accurate ratio of faeces:flotation solution. Thus, for all samples, the amount of faeces used was weighed exactly instead of using the measuring cone in the Fill‐FLOTAC device. In addition, the volume of saturated saline (prepared with a minimum specific gravity of 1.18) was measured using a 50 ml syringe for small volumes, or a graduated cylinder for larger volumes, instead of using the graduations on the Fill‐FLOTAC device.

A ‘wet’ preparation method was used for the composite samples in the BZ and LV groups to ensure complete homogenization of the sample. Individual samples were first kneaded by hand in the sample bags to break up faecal pellets and mix the sample thoroughly. Two grams of each homogenised individual sample in the treatment group was then weighed and placed in a large plastic bag. Saturated saline solution (360 ml; or 9 ml/g of faeces for treatment groups of <20 lambs) was added to the bag and the sample was homogenised well by kneading. The sealed bag was then inverted several times to disperse the eggs within the solution and an aliquot was immediately transferred to the Fill‐FLOTAC beaker, which was then used to fill the Mini‐FLOTAC slides in accordance with the standard Mini‐FLOTAC protocol.^28^, ^29^ The Day 14 MOX samples from three farms (farms 7, 9 and 12; Tables S1 and S2) were also prepared using this ‘wet’ method because their pre‐treatment (Day 0) FECs were <300 EPG and in one case because samples could not be processed individually within 7 days of collection. ‘Wet’ preparation of pooled faecal samples facilitates thorough mixing and is critical to accuracy, as a result of high levels of overdispersion in FEC across individuals within a group.^27^

A ‘dry’ preparation method was used for the individual samples in the IVM and MOX treatment groups, to obtain a representative aliquot of faeces from the whole sample. The whole individual sample was mixed in the sample bag by kneading. Two grams of the dry mixed sample was removed and weighed in the Fill‐FLOTAC beaker, and 18 ml of saturated saline solution was added. The sample was homogenised and the Mini‐FLOTAC slide was filled and eggs counted in accordance with the standard Mini‐FLOTAC protocol.^28^, ^29^ If less than 2 g of faeces was available the amount of saline solution was adjusted to maintain a 1 g:9 ml ratio.

### *H. contortus* identification

On Day 0, the saline/faeces suspension remaining from all egg counts for each farm was passed over a stack of 220, 150 and 38 μm sieves under running water. The sediment from the 38 μm (bottom) sieve was centrifuged for 2 minutes at 1500 RPM (400*g*), the supernatant discarded and the sediment re‐suspended in saturated saline solution to form a positive meniscus in the centrifuge tube. Eggs were collected by placing a coverslip on top of the meniscus for 10 minutes, then removing the coverslip in a smooth upward motion and washing eggs off the coverslip with water. The resulting egg suspension was adjusted to 500 eggs/ml of water by further sedimentation or dilution in water, as needed, in preparation for peanut agglutinin (PNA) staining.

The egg suspension was then thoroughly mixed and a 100 μl aliquot transferred into an Eppendorf tube with 100 μl PBS for storage in a freezer at −20°C. To prepare the sample for staining, 200 μl of working PNA solution was added (Vector Laboratories Catalogue No. FL‐1071):PBS solution (1:1000 v/v PNA:PBS) and vortex centrifuged at 6400*g* for 2 minutes. The supernatant was discarded, the sediment suspended in 1 ml of PBS and again centrifuged at 6400*g* for 2 minutes. The latter step was repeated a further three times to remove remaining PNA. Ten‐microlitre aliquots of the resulting suspension were viewed on a glass slide under a fluorescent compound microscope. First, eggs were identified under white light and then viewed under ultraviolet light at wavelength of 480 nm at higher power to determine whether the eggs fluoresced (*H. contortus*) or did not (other GIN species). At least 40 eggs were identified to determine the percentage of the population that comprised *H. contortus*.^30^ A control *H. contortus* sample was prepared and checked alongside every sample in the study to ensure the stain worked. Control eggs were collected from the faeces of an ewe that was examined postmortem, and in which 98% of adult GINs were identified as *H. contortus*.

Egg extraction and staining were repeated for each post‐treatment sample and treatment group if both *H. contortus* were identified in the Day 0 sample, and the post‐treatment reduction in FEC for a treatment group was <95%.

### Faecal egg count reduction calculations

Faecal egg count reduction percentage (FECR%) was calculated as (1 ‐ [FEC2/FEC1]) × 100 for the treatment groups where composite faecal samples were used, whereby FEC1 was the Day 0 FEC, and FEC2 was the Day 7 (LV) or Day 14 (BZ, IVM, MOX) FEC. For the treatment groups, AR was suspected if FECR% was <95%. A Bayesian method implemented in the *eggCounts* R package^31^, ^32^ was used to estimate mean FECR% and 95% confidence intervals for the IVM and MOX treatment groups where individual egg counts were available. For these treatment groups, AR was defined as a FECR% <95% with a lower confidence interval <90%, and suspected if only one of these conditions was met.^24^ All default parameters were used in the *fecr_stan* function of the *eggCounts* package, except where post‐treatment FECs were higher than pre‐treatment FECs, the prior distribution for *δ* was replaced with *list(priorDist = ‘normal’, hyperpars = c[1, 5])* to stabilise FECR% estimates, as described in the package documentation.^32^

FECR% was calculated for the undifferentiated egg counts (all trichostrongyle species) and also for *H. contortus* and ‘other’ trichostrongyle GIN species separately using the percentage *H. contortus* present before and after treatment to adjust the FECs.

## RESULTS

There was variation between treatment groups in mean pre‐treatment FEC on individual farms, even though lambs were systematically allocated to each of the treatment groups as described above, and the number of samples collected per treatment group exceeded the minimum recommendation for FECRT.^24^, ^33^ For example, mean pre‐treatment FEC in the four treatment groups on individual farms ranged as widely as 555–1539 EPG (farm 10; Table S1).

**FIGURE 1:**
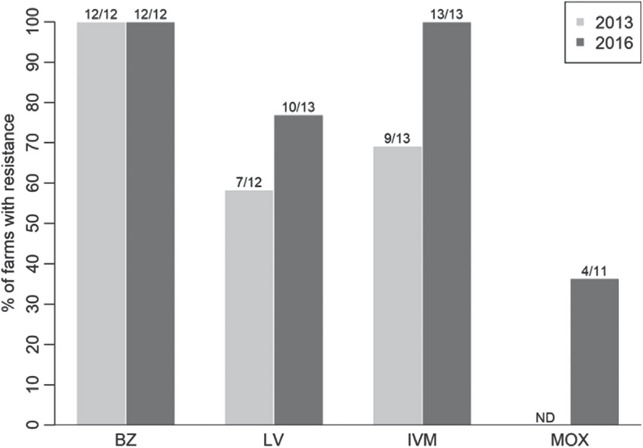
The percentage of farms with resistance or suspected resistance (where only one condition was satisfied or where 95% confidence intervals were not available) to the four anthelmintic treatments in 2013 (light grey^22^) and on the same farms in 2016 (dark grey). Moxidectin (MOX) resistance was not tested in 2013 (ND: not done). Numbers above the bars show the number of farms resistant (numerator) and the total number of farms tested (denominator)

**FIGURE 2:**
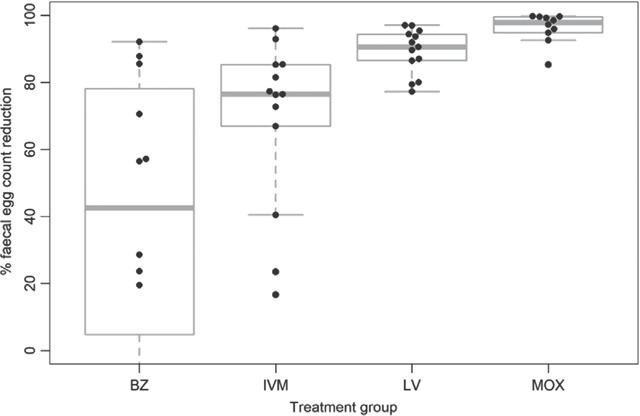
The distribution of faecal egg count reduction percentages (FECR%) on 13 farms for four treatment groups. BZ, benzimidazole; IVM, ivermectin; LV, levamisole; MOX, moxidectin. Note that the *y*‐axis has been truncated at zero for clarity. The FECR% was negative for the BZ treatment group on three farms and the lower whisker for the BZ boxplot extends to −95% (not visible). The FECR% was negative for the IVM treatment group on 2 farms (not visible). The dashed grey horizontal abline indicates the minimum FECR% threshold for efficacious treatment of 95%^24,33^

*H. contortus* eggs were identified by PNA staining in 85% of the pre‐treatment composite samples (11/13 farms) and comprised a median of 6% of eggs present in dung (range 1.6%–52%; Figure S1). Resistance (based on total egg counts) was suspected, but not confirmed due to lack of 95% confidence interval in composite FECR, to BZ on 100% (12/12) and to LV on 77% (10/13) of the farms; and suspected or confirmed to IVM on 100% (13/13) and to MOX on 36% (4/11) of the farms (Table 1). The percentage of farms with LV, IVM and MOX resistance was higher than FECRT results on the same farms 3 years previously (Figure 1). However, as different FEC methods were used in each year, statistical comparisons were not made. The average level of FEC reduction following treatment with each anthelmintic group followed a similar pattern in both years, being lowest (= highest level of resistance) for BZ, then IVM, LV and MOX (Figure 2).

**TABLE 1:** Summary of the faecal egg count reduction percentages (FECR%) estimated for the 13 farms

Treatment group	Species	Median FECR%	Minimum FECR%	Maximum FECR%	Farms confirmed resistant, % (*n*)	Farms suspected resistant, % (*n*)
BZ	All	42.5	−95.3	92.1	–	100% (12/12)
	*Haemonchus*	91.5	−209.3	100	–	50% (5/10)
	‘Other’ GIN	44.1	−108.3	88.5	–	100% (10/10)
LV	All	90.1	77.2	97.1	–	77% (10/13)
	*Haemonchus*	100	25.5	100	–	33% (3/9)
	‘Other’ GIN	86.7	57.0	95.7	–	88% (8/9)
IVM	All	76.5	−74.6	96.1	92% (12/13)	8% (1/13)
	*Haemonchus*	97.5	51.3	99.9	30% (3/10)	0%
	‘Other’ GIN	67.5	−116.3	82.0	100% (10/10)	0%
MOX	All	98.5	85.3	99.7	18% (2/11)	18% (2/11)
	*Haemonchus*	99.2	14.7	100	0%	20% (1/5)
	‘Other’ GIN	88.3	48.1	99.5	40% (2/5)	40% (2/5)

*Note*: Confirmed resistant % = percentage of farms with FECR% <95 and lower confidence interval (CI) <90; suspected resistant % = percentage of farms with FECR% <95 or lower CI <90, as per World Association for the Advancement of Veterinary Parasitology guidelines^24^; *n* is the number of farms.

Abbreviations: BZ, benzimidazole; GIN, gastrointestinal nematode; IVM, ivermectin; LV, levamisole; MOX, moxidectin.

**FIGURE 3:**
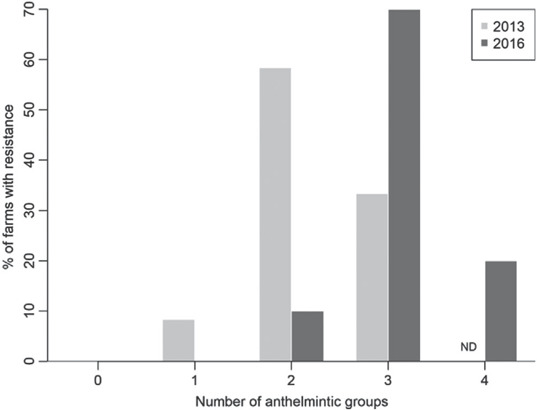
The percentage of farms with confirmed or suspected resistance to multiple anthelmintic groups tested in 2013 (light grey; *n* = 12), and on the same farms in 2016 (dark grey; *n* = 10). Farms where the full range of anthelmintic groups was not tested during the faecal egg count reduction test (FECRT), or where results are not available due to low pre‐treatment faecal egg counts (FECs) are not displayed (*n* = 1 in 2013; *n* = 3 in 2016). Moxidectin was not tested in 2013, therefore, the maximum possible anthelmintic groups in 2013 was three (ND: not done)

Overall, GINs were resistant, or suspected to be resistant, to a mode of three anthelmintic groups on each farm (70% of farms, *n* = 7/10; Figure 3). This was higher than in 2013, when GINs were resistant or suspected to be resistant to a mode of two anthelmintic groups per farm (58% of farms, *n* = 7/12). In the present study, 20% of farms were resistant to all anthelmintics tested (*n* = 2/10). None of the farms tested in 2016 had been susceptible to all anthelmintics in 2013.

**FIGURE 4:**
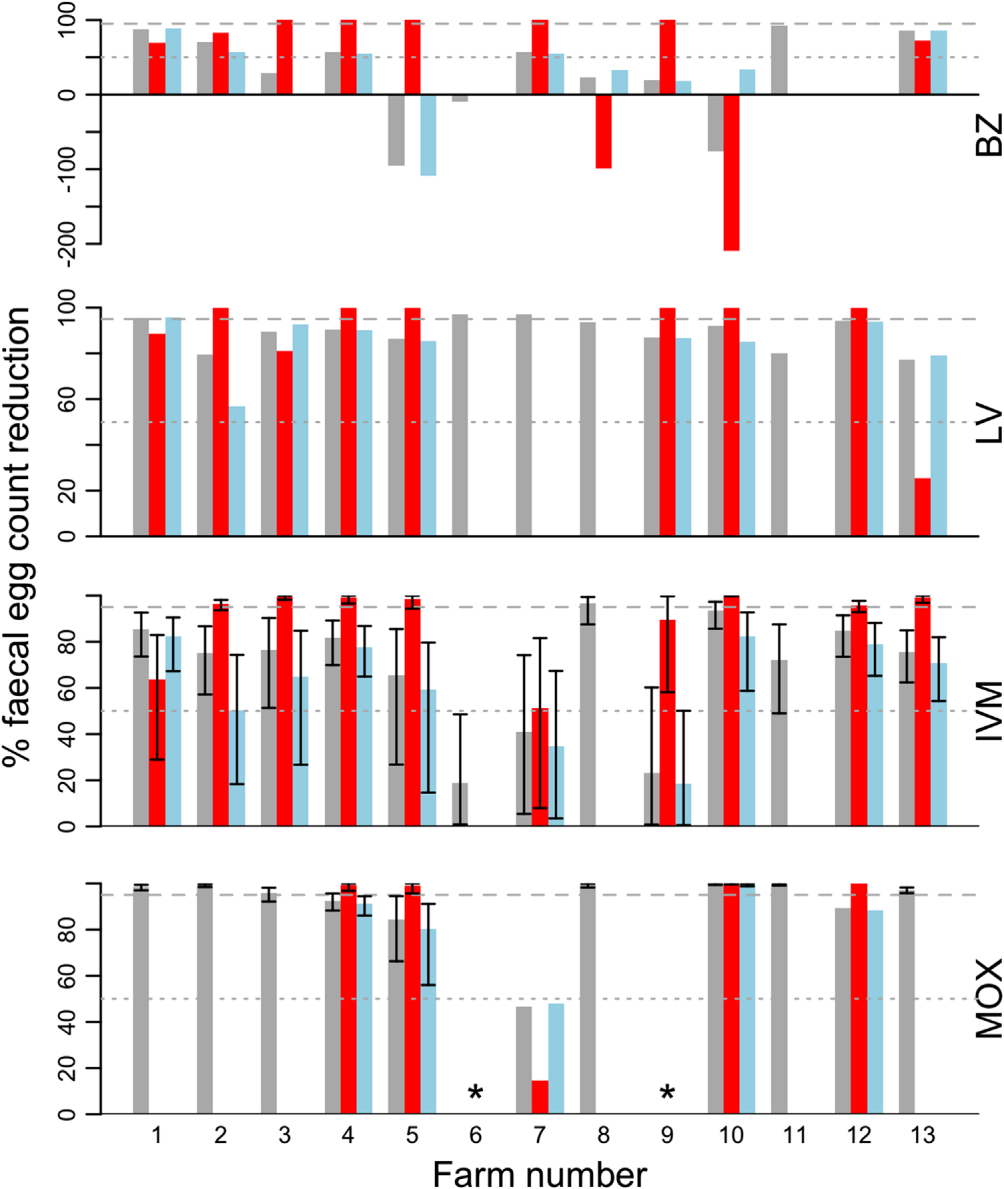
The estimated efficacy of benzimidazole (BZ), levamisole (LV), ivermectin (IVM) and moxidectin (MOX) on 13 farms, shown as percentage faecal egg count reduction (FECR%). Bars without 95% confidence intervals display FECR% estimated from composite faecal egg count (FEC). Bars with confidence intervals display Bayesian estimates of FECR% based on unpaired individual pre‐ and post‐treatment FEC. Efficacy against all gastrointestinal nematodes (GINs) present in the sample combined is shown in grey. The percentage of *Haemonchus contortus* eggs in the total sample was used to adjust the FECs to estimate efficacies for *H*. *contortus* (red) and other trichostrongyles (blue) separately. Note that the low (1.5%) BZ FECR% for other trichostrongyles for farm 3 is not visible at this scale. Where no red or blue bars are shown, this indicates that *H. contortus* was not present in the pre‐treatment sample, pre‐treatment FEC was too low, or the post‐treatment FEC was insufficient to allow differentiation. The dashed grey horizontal abline indicates the minimum percentage FEC reduction threshold for efficacious treatment of 95%.^24,33^ The dotted grey horizontal abline shows the 50% efficacy threshold, below which continued use of the anthelmintic is considered to lead to poor productivity and welfare.^8^ Data for the MOX treatment group for two farms (*) are not displayed as pre‐treatment FECs were low; for one other farm *H. contortus* eggs were not present, and for five other farms post‐treatment FEC were too low

Where *H. contortus* was present, anthelmintics tended to be more effective against *H. contortus* than against other GINs, based on FECR% (Figure 4), although the results were highly variable between farms. The estimated proportion of *H. contortus* eggs in FEC was low (<25%) on most of the farms (Figure S1; Table S1). Twenty‐five percent (*n* = 3) of farms had >25% *H. contortus* pre‐treatment. These same farms had >25% *H. contortus* post‐treatment in at least one treatment group.

The low *H. contortus* egg counts (EC × % *H. contortus* in the sample) presented difficulties in evaluating *H. contortus* FECR%. It was possible to assess *H. contortus* FECR% in the MOX treatment group on only 5/13 farms. On the others, *H. contortus* eggs were not present on two farms, Day 14 egg counts were too low to extract sufficient eggs from the samples for staining and specific identification (five farms), or Day 0 egg counts were too low to extract sufficient eggs (one farm).

## DISCUSSION

FECRTs were conducted on 13 farms in southwest England, to evaluate the efficacy of four anthelmintic groups—BZ, LV, IVM and MOX—as part of their ongoing veterinary care. These farms had previously conducted FECRT in 2013.^22^ Results demonstrate the diversity of AR profiles on farms, apparent progression of AR within a 3‐year period despite awareness by farmers, and the challenges of detecting AR in mixed GIN species infections.

Prevalence of AR among the farms tested was higher in 2016 than in 2013, despite strong advocacy for implementation of sustainable GIN control guidelines as set out by the Sustainable Control of Parasites in Sheep (SCOPS) initiative^8^, ^10^ by the veterinarians, demonstrating the potential speed at which resistance can develop. These findings are comparable to other AR surveys undertaken in the UK, Wales^16^ and Northern Ireland,^34^ suggesting that AR (according to current guidelines for its detection) is common throughout the UK.

The results for BZ were more variable than for the other anthelmintic classes, perhaps due to its prolonged historic use on farms. Lower levels of resistance were found to LV than IVM, which could be due to greater use of IVM, which is popular due to its activity against a wider range of parasites, including the scab mite *Psoroptes ovis*. In 2013, marginally fewer farms had LV resistance than IVM resistance but by 2016 this gap had widened.

Although resistance against multiple anthelmintic classes was prevalent in the sample population, the relative efficacy levels varied. Simply reporting ‘resistance’ to all three anthelmintic classes (BZ, LV, ML) may drive farmers to use new actives such as monepantel and derquantel (as a dual active with abamectin) inappropriately, whereas, based on the observed efficacy, some products may continue to be used appropriately. This is especially the case if FECRT methods make compromises on accuracy in the interests of practicality; for example, by excluding an untreated control group, as here, which reduces confidence in the result but is often necessary in order to be acceptable to farmers. We recommend that, where possible, anthelmintic efficacy is communicated in terms of FECR% and not only as a binary classification of resistant or not, to reduce the influence of statistical error on the result and to provide additional information that could help guide management. For example, although the prevalence of LV resistance was high in the present study (76.9% of the farms tested), efficacy in terms of FECR% has remained high relative to BZ and IVM, even on farms where it has fallen below the 95% threshold. It may be possible, with careful monitoring of pre‐ and post‐treatment FEC, to pragmatically use classes of anthelmintics where resistance has been confirmed but where efficacy remains relatively high (including the closely monitored use of sequentially administered combinations), to reduce reliance on ‘newer’ classes of anthelmintics, and therefore preserve their efficacy for as long as possible. However, this relies on high levels of compliance with FEC and other forms of monitoring (e.g. live weight gain) to ensure that productivity and welfare do not suffer. Further research, in collaboration with economists and social scientists with expertise in behaviour change, is needed to evaluate the feasibility of such approaches.

*H. contortus* eggs were present on 85% of the farms tested. This is higher than the averages of ∼50% of farms for the UK and ∼65% for England reported by Burgess et al.,^21^ lending weight to anecdotal reports that southwest England is a hotspot for *H. contortus* in the UK, and supporting mathematical model predictions of high climatic suitability for *H. contortus* in the southwest.^17^ Furthermore, *H. contortus* eggs remained in post‐treatment samples on seven of the 13 farms tested, suggesting potentially widespread AR in this species. The results should be treated with a degree of caution because the relatively low *Haemonchus*‐specific FEC inflate uncertainty in the genus‐specific FECR% estimates. Due to the small sample size in the present survey and the potential bias associated with the non‐random selection of farms, a wider survey to establish *H. contortus* prevalence on farms across southwest England would be beneficial for further insight. Nevertheless, this result is notable because, given the right conditions, *H. contortus* could increase rapidly in prevalence due to its high egg output,^15^ which has been proposed to underpin spread into temperate areas.^35^ Reports of anthelmintic‐resistant *H. contortus* are increasing throughout the world,^21^, ^36^, ^37^, ^38^, ^39^ and rising global temperatures could make conditions more favourable for this parasite to thrive and become even more widespread.^17^, ^40^ While such spread should be cause for concern, in the present study FECR% was often higher for *H. contortus* than for other genera, which does not support the theory that AR develops more quickly in this species due to its high fecundity.^41^ The development of AR at population level, therefore, might differ between areas where *H. contortus* is so far a minority species, and areas where it dominates.^36^ Neither inference can be made on any change in abundance of *H. contortus* over time on these particular farms, nor on its rate of AR development relative to other trichostrongylids, however, since species identification was not conducted in the earlier survey.^22^

Results from this survey have shown that, within mixed species populations, observed anthelmintic efficacy varies with the species present and with how FECRTs are analysed. As found in previous studies, not all species in a mixed infection may be resistant to the same extent^34^, ^42^ which presents potentially creative treatment options on farms living with resistance against multiple anthelmintic classes. For example, in this study BZ appeared to be 100% effective against *H. contortus* on farms 3 and 4, while BZ was less than 95% effective against other GIN species, in aggregate, on the same farms. BZ may therefore be an option for targeted treatment of haemonchosis on some farms, notwithstanding the risk of non‐target exposure of remaining GINs to BZ, as well as caution over the interpretation of the FECRT for *H. contortus*. On farm 3, for example, *H. contortus* comprised 27.5% of eggs in the pre‐treatment sample but this still yielded a pre‐treatment *H. contortus* FEC well below the recommended minimum required to detect AR (350 EPG × 0.275 = 96 EPG). A higher starting FEC would be beneficial to more accurately detect species‐specific AR in mixed infections, but this comes with an increased risk of compromised welfare. Nevertheless, these results offer an early indication of potential viable treatment options for haemonchosis even when AR against multiple anthelmintic classes is confirmed in mixed species infections. The species composition of mixed GIN infections on ovine farms therefore influences both the detection and management of AR, and differing seasonal profiles between species^40^ is likely to contribute to variation in FECRT results on farms within and between years. New methods to characterise species profiles at scale^43^ and at lower levels of resistance could help support earlier diagnosis of resistance and more nuanced approaches in future in research and practice.

The results for the BZ and LV treatment groups in this study are essentially a controlled ‘drench check’ as individual FECs were not conducted. After finding suspected AR using composite FEC, a controlled FECRT should ideally be completed on a farm‐by‐farm basis to ascertain which classes of anthelmintics are useful for control of haemonchosis and other GIN species. Future work would benefit from identifying ‘other’ strongyle species to determine efficacy of anthelmintics further. McIntyre et al.^44^, for instance, found the presence of a dual IVM and BZ‐resistant *T. circumcincta* population, which was not evident on undifferentiated FECRT. Species profiles vary widely within as well as between farms by season and stock class,^45^ and this is likely to affect FECRT results.

Results of this study are not truly representative of the AR situation in the region, since farms were selected based on previous doubts over anthelmintic efficacy. Representative studies of AR are especially difficult given their voluntary nature and the high level of commitment required by study farms.^46^ Nevertheless, they provide an insight into how quickly AR can progress by comparing the same farms over a 3‐year period. Responsible use of anthelmintics is essential to increase the longevity of these products and farmers need to be encouraged to adopt the SCOPS guidelines in their management. The diversity of AR profiles observed in this study, and potential implications for GIN control, supports proposed restrictions on advertising of anthelmintics, in order to encourage careful context‐specific anthelmintic selection. Routine FEC and early FECRT (before resistance is suspected and performance suffers) should be used as a resistance monitoring tool, to maintain sustainability of anthelmintics by choosing the right product and time to treat.

## ETHICS STATEMENT

The FECRTs in the present study were conducted by veterinarians and trained technicians (Torch Farm & Equine, UK) for clinical purposes, to inform effective GIN control due to an apparent increase in haemonchosis in the region in recent years. Anonymised samples and data were shared with researchers at the University of Bristol for analysis, with informed consent of the farmers.

## AUTHOR CONTRIBUTIONS

Mike J. Glover was responsible for organising and sampling both the 2013 and 2016 studies. Eric R. Morgan, Hannah Rose Vineer and Katie Bull assisted Mike J. Glover in the 2016 sample collection. Hannah Rose Vineer and Katie Bull processed the lab samples and all authors contributed to the manuscript.

## CONFLICT OF INTEREST

The authors declare no conflicts of interest.

## Supporting information

Supporting Information

## Data Availability

The data that supports the findings of this study are available in the supplementary material of this article.
