# Supplementary material for: Increasing resistance to multiple anthelmintic classes in gastrointestinal nematodes on sheep farms in southwest England
Source: Vet Rec. 2022 Mar 26;190(11):e1531. doi: 10.1002/vetr.1531 (PMC9310741; doi:10.1002/vetr.1531)
Supplement: Supplementary file 1 — Supporting Information [file VETR-190-no-s001.docx]

**SUPPLEMENTARY DATA**

**
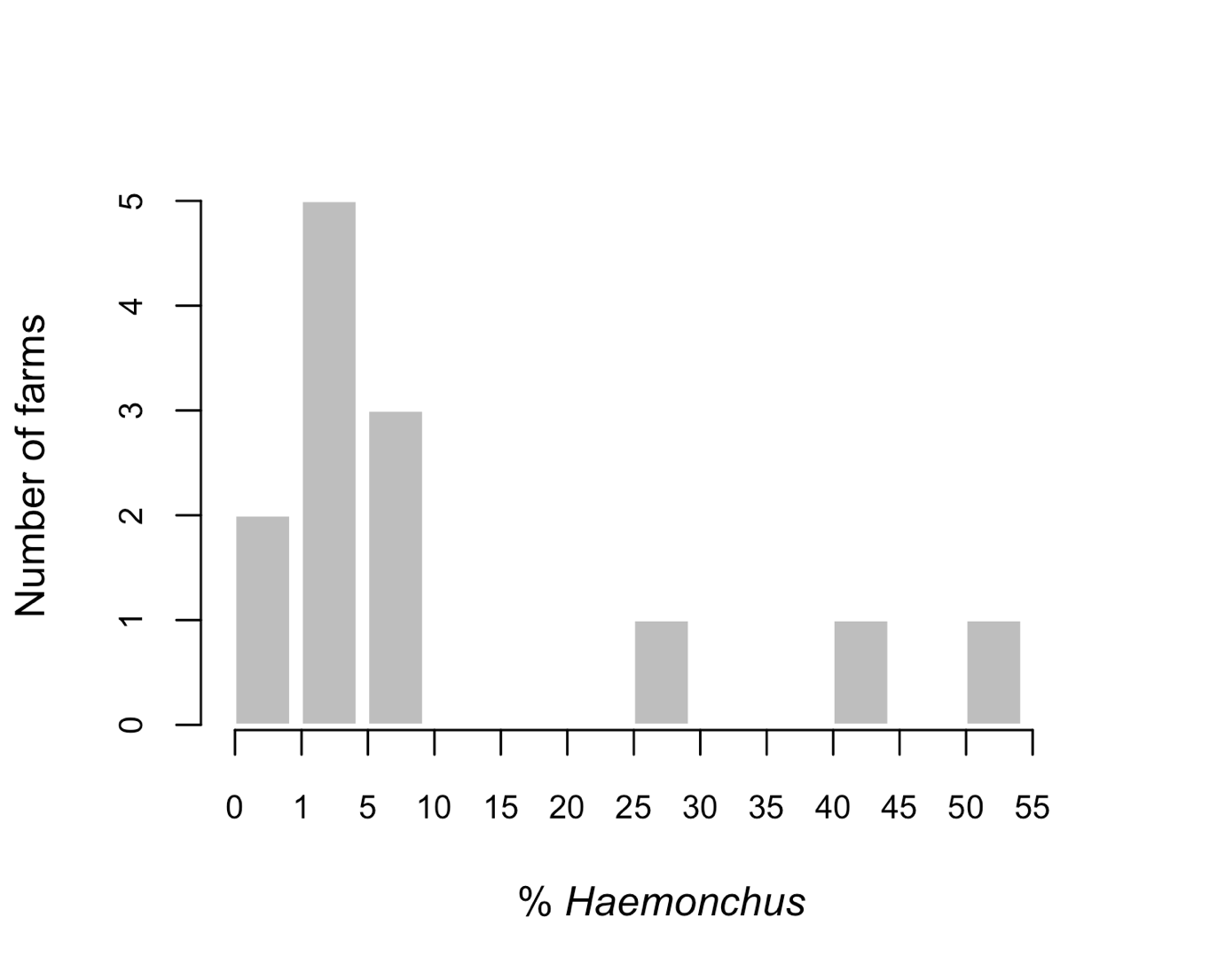
**

**Figure S1.** The distribution of *Haemonchus contortus* eggs in the flocks sampled as a percentage of total eggs in dung. *Haemonchus contortus* eggs comprised a median of 6% of eggs in composite samples submitted by the farms. NB 3 of the 13 farms had more than 25% *H.contortus* in their pre-treatment sample.

**Table S1.** Faecal egg counts and proportion of *Haemonchus contortus* present pre-treatment and post-treatment. FEC = faecal egg count, FECR% = percent reduction in FEC, epg = eggs per gram of faeces, BZ = benzimidazole, LV = levamisole, IVM = ivermectin, MOX = moxidectin. No BZ group was included on farm 12 due to lack of lambs available for sampling.

| Farm |  | **BZ*** | | **LV*** | | **IVM** | | **MOX** | |
| --- | --- | --- | --- | --- | --- | --- | --- | --- | --- |
|  |  | Pre | Post | Pre | Post | Pre | Post | Pre | Post |
| **1** | **FEC (epg)** | 775 | 95 | 760 | 35 | 854 | 108 | 662 | 9.12 |
|  | **FECR%** |  | 87.7 |  | 95.4 |  | 85.0 (71.9 – 92.7) |  | 98.5 (97.0 – 99.3) |
|  | **% *Haemonchus*** | 4 | 10 | 4 | 10 | 4 | 10 | 4 | - |
|  | **FECR% *Haemonchus*** |  | 69.4 |  | 88.5 |  | 64.8 (36.0 – 82.0) |  | - |
|  | **FECR% other** |  | 88.5 |  | 95.7 |  | 82.0 (67.4 – 90.7) |  | - |
| **2** | **FEC (epg)** | 1240 | 365 | 1210 | 250 | 761.5 | 165 | 945.75 | 5.75 |
|  | **FECR%** |  | 70.6 |  | 79.3 |  | 76.3 (59.1 – 86.9) |  | 99.2 (98.3-99.6) |
|  | **% *Haemonchus*** | 52 | 30 | 52 | 0 | 52 | 7 | 52 | - |
|  | **FECR% *Haemonchus*** |  | 83.0 |  | 100 |  | 96.5 (94.0 – 98.1) |  | - |
|  | **FECR% other** |  | 57.1 |  | 57 |  | 47.2 (16.1 – 71.7) |  | - |
| **3** | **FEC (epg)** | 350 | 250 | 240 | 25 | 212.5 | 40.75 | 245.3 | 8.95 |
|  | **FECR%** |  | 28.6 |  | 89.6 |  | 77.3 (55.7 – 90.5) |  | 95.9 (91.9 – 98.1) |
|  | **% *Haemonchus*** | 27.5 | 0 | 27.5 | 50 | 27.5 | 0 | 27.5 | - |
|  | **FECR% *Haemonchus*** |  | 100 |  | 81.1 |  | 99.5 (98.2 – 100) |  | - |
|  | **FECR% other** |  | 1.5 |  | 92.8 |  | 64.3 (29.3 – 83.8) |  | - |
| **4** | **FEC (epg)** | 525 | 225 | 1055 | 100 | 654.75 | 110 | 643.75 | 44.5 |
|  | **FECR%** |  | 57.1 |  | 90.5 |  | 81.5 (69.7 – 89.0) |  | 92.5 (87.8 – 95.8) |
|  | **% *Haemonchus*** | 5 | 0 | 5 | 0 | 5 | 0 | 5 | 0 |
|  | **FECR% *Haemonchus*** |  | 100 |  | 100 |  | 99.2 (96.8 – 100) |  | 99.2 (96.7 – 100) |
|  | **FECR% other** |  | 54.9 |  | 90 |  | 78.0 (66.0 – 87.1) |  | 91.1 (86.1 – 94.5) |
| **5** | **FEC (epg)** | 320 | 625 | 480 | 65 | 293.75 | 82.75 | 376.5 | 45.5 |
|  | **FECR%** |  | -95.3 |  | 86.5 |  | 66.9 (34.3 – 86.0) |  | 85.3 (66.3 – 94.5) |
|  | **% *Haemonchus*** | 6.25 | 0 | 6.25 | 0 | 6.25 | 0 | 6.25 | 0 |
|  | **FECR% *Haemonchus*** |  | 100 |  | 100 |  | 98.4 (94.1 – 100) |  | 98.9 (95.4 – 100) |
|  | **FECR% other** |  | -108.3 |  | 85.6 |  | 56 (14.5-80.9) |  | 80.59 (59.9-91.4) |
| **6** | **FEC (epg)** | 250 | 275 | 170 | 5 | 153.8 | 197 | 84 | 4 |
|  | **FECR%** |  | -10.0 |  | 97.1 |  | 16.6 (0.9 – 46.1) |  | ND |
|  | **% *Haemonchus*** | 0 | - | 0 | - | 0 | - | 0 | - |
|  | **FECR% *Haemonchus*** |  | - |  | - |  | - |  | - |
|  | **FECR% other** |  | - |  | - |  | - |  | - |
| **7** | **FEC (epg)** | 195 | 85 | 165 | 5 | 180 | 101.11 | 150* | 80* |
|  | **FECR%** |  | 56.4 |  | 97.0 |  | 40.4 (4.9 – 71.3) |  | 46.7 |
|  | **% *Haemonchus*** | 4.17 | 0 | 4.17 | - | 4.17 | 4 | 4.17 | 6.67 |
|  | **FECR% *Haemonchus*** |  | 100 |  | - |  | 51.3 (7.5 – 82.2) |  | 14.7 |
|  | **FECR% other** |  | 54.5 |  | - |  | 37.04 (4.4-69.5) |  | 48 |
| **8** | **FEC (epg)** | 360 | 275 | 555 | 35 | 652 | 10.75 | 486 | 0.79 |
|  | **FECR%** |  | 23.6 |  | 93.7 |  | 96.1 (84.5 – 99.4) |  | 99.6 (98.4 – 100) |
|  | **% *Haemonchus*** | 6.98 | 18.18 | 6.98 | - | 6.98 | - | 6.98 | - |
|  | **FECR% *Haemonchus*** |  | -99.0 |  | - |  | - |  | - |
|  | **FECR% other** |  | 32.8 |  | - |  | - |  | - |
| **9** | **FEC (epg)** | 205 | 165 | 115 | 15 | 181.05 | 245.79 | 25* | 25* |
|  | **FECR%** |  | 19.5 |  | 87.0 |  | 23.5 (0.9 – 58.1) |  | ND |
|  | **% *Haemonchus*** | 1.61 | 0 | 1.61 | 0 | 1.61 | 0 | 1.61 | 0 |
|  | **FECR% *Haemonchus*** |  | 100 |  | 100 |  | 90.0 (60.1 – 99.7) |  | ND |
|  | **FECR% other** |  | 18.2 |  | 86.7 |  | 16.72 (0.4-4) |  | - |
| **10** | **FEC (epg)** | 800 | 1405 | 740 | 60 | 1539.25 | 74.75 | 554.75 | 1 |
|  | **FECR%** |  | -75.6 |  | 91.9 |  | 92.3 (83.6 – 97.2) |  | 99.7 (99.4 – 99.9) |
|  | **% *Haemonchus*** | 45 | 79.25 | 45 | 0 | 45 | 0 | 45 | 0 |
|  | **FECR% *Haemonchus*** |  | -209.3 |  | 100 |  | 99.9 (99.7 – 100) |  | 99.9 (99.5 – 100) |
|  | **FECR% other** |  | 33.7 |  | 85.2 |  | 83.1 (63.4-92.7) |  | 99.5 (98.7-99.9) |
| **11** | **FEC (epg)** | 695 | 55 | 650 | 130 | 524 | 126.5 | 636.5 | 2.5 |
|  | **FECR%** |  | 92.1 |  | 80.0 |  | 72.7 (51.4 – 86.9) |  | 99.5 (99.1 – 99.8) |
|  | **% *Haemonchus*** | 0 | - | 0 | - | 0 | - | 0 | - |
|  | **FECR% *Haemonchus*** |  | - |  | - |  | - |  | - |
|  | **FECR% other** |  | - |  | - |  | - |  | - |
| **12** | **FEC (epg)** |  |  | 1420 | 80 | 1359 | 172.5 | 1490.5 | 160* |
|  | **FECR%** |  |  |  | 94.4 |  | 85.3 (75.7 – 91.6) |  | 89.3 |
|  | **% *Haemonchus*** |  |  | 8.5 | 0 | 8.5 | 2.43 | 8.5 | 0 |
|  | **FECR% *Haemonchus*** |  |  |  | 100 |  | 95.7 (92.9 – 97.7) |  | 100 |
|  | **FECR% other** |  |  |  | 93.8 |  | 79.9 (66.9-87.7) |  | 88.3 |
| **13** | **FEC (epg)** | 900 | 130 | 855 | 195 | 953 | 182.78 | 1141 | 29 |
|  | **FECR%** |  | 85.6 |  | 77.2 |  | 76.5 (62.5 – 86.1) |  | 97.2 (95.6 – 98.3) |
|  | **% *Haemonchus*** | 3.5 | 6.67 | 3.5 | 11.43 | 3.5 | 0 | 3.5 | - |
|  | **FECR% *Haemonchus*** |  | 72.5 |  | 25.5 |  | 99.2 (96.8 – 100) |  | - |
|  | **FECR% other** |  | 86.0 |  | 79.1 |  | 70.61 (54.5-82.2) |  | - |

*composite FEC

ND = FECR% not calculated due to low pre-treatment FEC

- = Efficacy was above 95% or no *Haemonchus contortus* present in the pre-treatment sample, therefore staining was not necessary

**Table S2**. Individual faecal egg counts (FEC) for lambs in the ivermectin (IVM) and moxidectin (MOX) treatment groups. Note that lambs were not individually identified and therefore FEC are not paired.

|  | **IVM** | | | | **MOX** | | | |
| --- | --- | --- | --- | --- | --- | --- | --- | --- |
|  | **Pre** | | **Post** | | **Pre** | | **Post** | |
| **Farm** | **Trichostrongyles** | ***Nematodirus*** | **Trichostrongyles** | ***Nematodirus*** | **Trichostrongyles** | ***Nematodirus*** | **Trichostrongyles** | ***Nematodirus*** |
|  |  |  |  |  |  |  |  |  |
| 1 | 440 | 0 | 15 | 0 | 405 | 30 | 0 | 0 |
| 1 | 1575 | 430 | 940 | 0 | 195 | 70 | 20 | 0 |
| 1 | 970 | 100 | 45 | 0 | 960 | 65 | 5 | 0 |
| 1 | 2675 | 20 | 60 | 0 | 375 | 105 | 10 | 0 |
| 1 | 380 | 10 | 70 | 0 | 310 | 205 | 0 | 0 |
| 1 | 915 | 140 | 50 | 0 | 470 | 70 | 0 | 0 |
| 1 | 285 | 220 | 35 | 0 | 200 | 5 | 0 | 0 |
| 1 | 1080 | 35 | 40 | 0 | 2080 | 250 | 0 | 0 |
| 1 | 495 | 190 | 105 | 0 | 720 | 355 | 0 | 0 |
| 1 | 540 | 0 | 70 | 0 | 855 | 365 | 20 | 0 |
| 1 | 995 | 20 | 5 | 0 | 830 | 10 | 5 | 0 |
| 1 | 665 | 165 | 15 | 0 | 990 | 60 | 25 | 0 |
| 1 | 995 | 10 | 10 | 0 | 2505 | 135 | 5 | 0 |
| 1 | 850 | 65 | 0 | 0 | 340 | 10 | 45 | 0 |
| 1 | 345 | 150 | 45 | 0 | 535 | 80 | 5 | 0 |
| 1 | 890 | 0 | 130 | 0 | 435 | 90 | 0 | 0 |
| 1 | 210 | 5 | 235 | 0 | 110 | 10 | 15 | 0 |
| 1 | 1310 | 45 | 80 | 0 | 460 | 80 |  |  |
| 1 | 530 | 20 |  |  | 280 | 55 |  |  |
| 1 | 940 | 75 |  |  | 200 | 105 |  |  |
|  |  |  |  |  |  |  |  |  |
| 2 | 1115 | 55 | 380 | 0 | 965 | 10 | 5 | 0 |
| 2 | 1055 | 0 | 125 | 0 | 2140 | 5 | 0 | 0 |
| 2 | 510 | 40 | 235 | 0 | 770 | 40 | 0 | 0 |
| 2 | 100 | 0 | 600 | 0 | 795 | 85 | 0 | 0 |
| 2 | 305 | 0 | 70 | 0 | 425 | 20 | 0 | 0 |
| 2 | 885 | 10 | 130 | 0 | 1025 | 25 | 0 | 0 |
| 2 | 1700 | 40 | 165 | 0 | 2230 | 10 | 0 | 0 |
| 2 | 2900 | 20 | 485 | 0 | 385 | 30 | 10 | 0 |
| 2 | 175 | 60 | 75 | 0 | 800 | 20 | 0 | 0 |
| 2 | 2165 | 35 | 25 | 0 | 1380 | 25 | 0 | 0 |
| 2 | 225 | 0 | 5 | 0 | 505 | 5 | 0 | 0 |
| 2 | 570 | 25 | 45 | 0 | 3115 | 65 | 0 | 0 |
| 2 | 390 | 35 | 100 | 0 | 255 | 10 | 35 | 0 |
| 2 | 375 | 15 | 40 | 0 | 250 | 75 | 0 | 0 |
| 2 | 405 | 25 | 215 | 0 | 1650 | 35 | 5 | 0 |
| 2 | 275 | 15 | 135 | 0 | 200 | 25 | 0 | 0 |
| 2 | 710 | 5 | 10 | 0 | 475 | 30 | 0 | 0 |
| 2 | 450 | 0 | 265 | 0 | 405 | 25 | 0 | 0 |
| 2 | 200 | 0 | 55 | 0 | 175 | 0 | 60 | 0 |
| 2 | 715 | 20 | 140 | 0 | 970 | 10 | 5 | 0 |
|  |  |  |  |  |  |  |  |  |
| 3 | 705 | 0 | 145 | 0 | 245 | 0 | 50 | 0 |
| 3 | 50 | 0 | 30 | 0 | 285 | 0 | 10 | 0 |
| 3 | 115 | 20 | 5 | 0 | 460 | 0 | 20 | 0 |
| 3 | 65 | 0 | 15 | 0 | 540 | 10 | 20 | 0 |
| 3 | 275 | 0 | 55 | 0 | 85 | 0 | 30 | 0 |
| 3 | 165 | 0 | 0 | 0 | 200 | 10 | 0 | 0 |
| 3 | 130 | 5 | 20 | 0 | 300 | 0 | 0 | 0 |
| 3 | 225 | 0 | 50 | 0 | 175 | 0 | 15 | 0 |
| 3 | 60 | 0 | 0 | 0 | 395 | 0 | 0 | 0 |
| 3 | 540 | 0 | 0 | 0 | 295 | 40 | 0 | 0 |
| 3 | 165 | 5 | 0 | 0 | 170 | 20 | 0 | 0 |
| 3 | 60 | 5 | 0 | 0 | 15 | 0 | 0 | 0 |
| 3 | 30 | 0 | 30 | 0 | 170 | 0 | 0 | 0 |
| 3 | 125 | 0 | 105 | 0 | 200 | 0 | 10 | 0 |
| 3 | 60 | 0 | 15 | 0 | 460 | 0 | 0 | 0 |
| 3 | 160 | 0 | 75 | 0 | 485 | 5 | 0 | 0 |
| 3 | 335 | 10 | 0 | 0 | 195 | 5 | 15 | 0 |
| 3 | 305 | 195 | 0 | 0 | 55 | 5 | 0 | 0 |
| 3 | 290 | 10 | 0 | 0 | 40 | 0 | 0 | 0 |
| 3 | 390 | 25 | 270 | 0 | 135 | 10 |  |  |
|  |  |  |  |  |  |  |  |  |
| 4 | 345 | 55 | 115 | 0 | 450 | 40 | 60 | 5 |
| 4 | 1105 | 65 | 70 | 0 | 570 | 75 | 125 | 0 |
| 4 | 695 | 5 | 340 | 0 | 625 | 25 | 25 | 0 |
| 4 | 850 | 90 | 215 | 0 | 475 | 75 | 30 | 0 |
| 4 | 1135 | 40 | 110 | 0 | 450 | 65 | 150 | 0 |
| 4 | 320 | 90 | 5 | 0 | 735 | 90 | 15 | 0 |
| 4 | 240 | 55 | 70 | 0 | 300 | 100 | 10 | 0 |
| 4 | 1210 | 85 | 65 | 0 | 540 | 110 | 20 | 0 |
| 4 | 375 | 0 | 155 | 0 | 485 | 75 | 10 | 0 |
| 4 | 855 | 45 | 100 | 0 | 1215 | 30 | 0 | 0 |
| 4 | 1450 | 135 | 200 | 0 | 510 | 55 | 25 | 0 |
| 4 | 1485 | 175 | 165 | 0 | 640 | 80 | 5 | 0 |
| 4 | 245 | 125 | 320 | 0 | 915 | 25 | 110 | 0 |
| 4 | 335 | 120 | 55 | 0 | 610 | 30 | 20 | 0 |
| 4 | 220 | 90 | 30 | 0 | 800 | 130 | 25 | 0 |
| 4 | 875 | 35 | 105 | 0 | 760 | 55 | 30 | 0 |
| 4 | 685 | 55 | 5 | 0 | 405 | 35 | 35 | 0 |
| 4 | 165 | 70 | 35 | 0 | 660 | 145 | 165 | 0 |
| 4 | 415 | 55 | 20 | 0 | 545 | 145 | 30 | 0 |
| 4 | 90 | 5 | 20 | 0 | 1185 | 45 | 0 | 0 |
|  |  |  |  |  |  |  |  |  |
| 5 | 595 | 20 | 0 | 0 | 370 | 60 | 25 | 0 |
| 5 | 285 | 0 | 30 | 0 | 875 | 10 | 535 | 0 |
| 5 | 440 | 0 | 25 | 0 | 250 | 0 | 0 | 0 |
| 5 | 60 | 0 | 20 | 0 | 95 | 5 | 10 | 0 |
| 5 | 110 | 0 | 20 | 0 | 85 | 0 | 0 | 0 |
| 5 | 155 | 0 | 0 | 0 | 165 | 5 | 5 | 0 |
| 5 | 125 | 0 | 15 | 0 | 220 | 0 | 15 | 0 |
| 5 | 115 | 0 | 25 | 0 | 90 | 0 | 15 | 0 |
| 5 | 40 | 0 | 15 | 0 | 210 | 0 | 15 | 0 |
| 5 | 280 | 0 | 5 | 0 | 310 | 0 | 5 | 0 |
| 5 | 20 | 0 | 25 | 0 | 780 | 0 | 15 | 0 |
| 5 | 230 | 0 | 85 | 0 | 210 | 0 | 0 | 0 |
| 5 | 740 | 90 | 760 | 0 | 180 | 0 | 0 | 0 |
| 5 | 250 | 0 | 25 | 0 | 630 | 0 | 165 | 0 |
| 5 | 130 | 0 | 15 | 0 | 430 | 0 | 0 | 0 |
| 5 | 910 | 50 | 10 | 0 | 680 | 0 | 10 | 0 |
| 5 | 100 | 0 | 0 | 0 | 1240 | 135 | 5 | 0 |
| 5 | 470 | 10 | 520 | 0 | 195 | 0 | 85 | 0 |
| 5 | 70 | 0 | 45 | 0 | 270 | 0 | 5 | 0 |
| 5 | 750 | 10 | 15 | 0 | 245 | 10 | 0 | 0 |
|  |  |  |  |  |  |  |  |  |
| 6 | 5 | 20 | 225 | 0 | 25 | 10 | 0 | 0 |
| 6 | 70 | 25 | 60 | 0 | 590 | 25 | 0 | 0 |
| 6 | 20 | 10 | 185 | 0 | 140 | 0 | 25 | 0 |
| 6 | 110 | 10 | 385 | 0 | 55 | 15 | 5 | 0 |
| 6 | 90 | 25 | 450 | 0 | 40 | 15 | 0 | 0 |
| 6 | 100 | 10 | 330 | 0 | 65 | 0 | 5 | 0 |
| 6 | 60 | 5 | 310 | 0 | 170 | 20 | 0 | 0 |
| 6 | 1260 | 0 | 125 | 0 | 25 | 5 | 10 | 0 |
| 6 | 165 | 0 | 165 | 0 | 50 | 5 | 0 | 0 |
| 6 | 90 | 0 | 25 | 0 | 55 | 10 | 0 | 0 |
| 6 | 195 | 40 | 115 | 0 | 40 | 15 | 5 | 0 |
| 6 | 210 | 15 | 50 | 0 | 20 | 20 | 0 | 0 |
| 6 | 75 | 0 | 60 | 0 | 105 | 5 | 10 | 0 |
| 6 | 90 | 15 | 255 | 0 | 50 | 10 | 0 | 0 |
| 6 | 270 | 10 | 215 | 0 | 30 | 5 | 0 | 0 |
| 6 | 10 | 15 |  |  | 15 | 15 |  |  |
| 6 | 90 | 10 |  |  | 75 | 0 |  |  |
| 6 | 30 | 5 |  |  | 35 | 35 |  |  |
| 6 | 35 | 0 |  |  | 55 | 5 |  |  |
| 6 | 100 | 5 |  |  | 40 | 0 |  |  |
|  |  |  |  |  |  |  |  |  |
| 7 | 5 | 0 | 435 | 0 |  |  |  |  |
| 7 | 155 | 0 | 25 | 0 |  |  |  |  |
| 7 | 35 | 0 | 25 | 0 |  |  |  |  |
| 7 | 20 | 70 | 0 | 0 |  |  |  |  |
| 7 | 45 | 0 | 170 | 0 |  |  |  |  |
| 7 | 70 | 0 | 65 | 0 |  |  |  |  |
| 7 | 190 | 0 | 135 | 0 |  |  |  |  |
| 7 | 60 | 0 | 40 | 0 |  |  |  |  |
| 7 | 0 | 0 | 170 | 0 |  |  |  |  |
| 7 | 130 | 115 | 30 | 0 |  |  |  |  |
| 7 | 770 | 0 | 55 | 0 |  |  |  |  |
| 7 | 0 | 0 | 35 | 0 |  |  |  |  |
| 7 | 250 | 40 | 50 | 0 |  |  |  |  |
| 7 | 490 | 0 | 25 | 0 |  |  |  |  |
| 7 | 65 | 5 | 40 | 0 |  |  |  |  |
| 7 | 285 | 0 | 65 | 0 |  |  |  |  |
| 7 | 90 | 0 | 45 | 0 |  |  |  |  |
| 7 | 560 | 115 | 410 | 0 |  |  |  |  |
| 7 | 0 | 0 |  |  |  |  |  |  |
| 7 | 390 | 0 |  |  |  |  |  |  |
|  |  |  |  |  |  |  |  |  |
| 8 | 275 | 10 | 0 | 0 | 105 | 0 | 0 | 0 |
| 8 | 1410 | 0 | 0 | 0 | 40 | 0 | 0 | 0 |
| 8 | 390 | 0 | 0 | 0 | 0 | 0 | 0 | 0 |
| 8 | 0 | 0 | 20 | 0 | 430 | 0 | 5 | 0 |
| 8 | 2130 | 30 | 0 | 0 | 0 | 0 | 5 | 0 |
| 8 | 1060 | 5 | 0 | 0 | 2300 | 100 | 0 | 0 |
| 8 | 10 | 35 | 0 | 0 | 30 | 20 | 0 | 0 |
| 8 | 610 | 0 | 25 | 0 | 10 | 0 | 0 | 0 |
| 8 | 2980 | 0 | 0 | 0 | 1990 | 115 | 0 | 0 |
| 8 | 1400 | 150 | 10 | 0 | 1670 | 30 | 0 | 0 |
| 8 | 740 | 10 | 115 | 0 | 0 | 0 | 5 | 0 |
| 8 | 0 | 0 | 10 | 0 | 420 | 0 | 0 | 0 |
| 8 | 0 | 100 | 0 | 0 | 90 | 0 | 0 | 0 |
| 8 | 30 | 40 | 0 | 0 | 25 | 0 | 0 | 0 |
| 8 | 610 | 0 | 5 | 0 | 80 | 0 | 0 | 0 |
| 8 | 0 | 0 | 0 | 0 | 20 | 0 | 0 | 0 |
| 8 | 640 | 40 | 5 | 0 | 1790 | 65 | 0 | 0 |
| 8 | 120 | 25 | 0 | 0 | 0 | 0 | 0 | 0 |
| 8 | 0 | 0 | 15 | 0 | 720 | 10 | 0 | 0 |
| 8 |  |  | 10 | 0 |  |  |  |  |
|  |  |  |  |  |  |  |  |  |
| 9 | 230 | 0 | 30 | 0 |  |  |  |  |
| 9 | 10 | 0 | 15 | 0 |  |  |  |  |
| 9 | 0 | 0 | 700 | 0 |  |  |  |  |
| 9 | 830 | 145 | 590 | 0 |  |  |  |  |
| 9 | 125 | 25 | 65 | 0 |  |  |  |  |
| 9 | 100 | 65 | 5 | 0 |  |  |  |  |
| 9 | 40 | 15 | 30 | 0 |  |  |  |  |
| 9 | 0 | 0 | 445 | 0 |  |  |  |  |
| 9 | 280 | 220 | 10 | 0 |  |  |  |  |
| 9 | 5 | 10 | 0 | 0 |  |  |  |  |
| 9 | 25 | 60 | 45 | 0 |  |  |  |  |
| 9 | 5 | 0 | 1370 | 0 |  |  |  |  |
| 9 | 0 | 5 | 275 | 0 |  |  |  |  |
| 9 | 190 | 0 | 510 | 0 |  |  |  |  |
| 9 | 0 | 0 | 15 | 0 |  |  |  |  |
| 9 | 520 | 0 | 5 | 0 |  |  |  |  |
| 9 | 290 | 0 | 50 | 0 |  |  |  |  |
| 9 | 310 | 0 | 135 | 0 |  |  |  |  |
| 9 | 455 | 0 | 375 | 0 |  |  |  |  |
|  |  |  |  |  |  |  |  |  |
| 10 | 360 | 0 | 280 | 0 | 355 | 5 | 5 | 0 |
| 10 | 870 | 0 | 15 | 0 | 180 | 0 | 0 | 0 |
| 10 | 80 | 0 | 30 | 0 | 1400 | 0 | 0 | 0 |
| 10 | 3860 | 0 | 5 | 0 | 775 | 0 | 0 | 0 |
| 10 | 2920 | 0 | 60 | 0 | 685 | 0 | 0 | 0 |
| 10 | 265 | 0 | 50 | 0 | 1575 | 0 | 0 | 0 |
| 10 | 120 | 0 | 85 | 0 | 95 | 0 | 0 | 0 |
| 10 | 285 | 0 | 10 | 0 | 235 | 0 | 0 | 0 |
| 10 | 350 | 0 | 20 | 0 | 620 | 0 | 0 | 0 |
| 10 | 6270 | 0 | 20 | 0 | 1115 | 0 | 0 | 0 |
| 10 | 100 | 0 | 0 | 0 | 340 | 0 | 0 | 0 |
| 10 | 60 | 0 | 55 | 0 | 1140 | 0 | 15 | 0 |
| 10 | 7360 | 0 | 190 | 0 | 295 | 0 | 0 | 0 |
| 10 | 465 | 0 | 10 | 0 | 150 | 0 | 0 | 0 |
| 10 | 2490 | 25 | 0 | 0 | 25 | 0 | 0 | 0 |
| 10 | 1525 | 5 | 15 | 0 | 440 | 0 | 0 | 0 |
| 10 | 500 | 0 | 10 | 0 | 80 | 0 | 0 | 0 |
| 10 | 410 | 0 | 0 | 0 | 310 | 0 | 0 | 0 |
| 10 | 405 | 0 | 515 | 0 | 70 | 0 | 0 | 0 |
| 10 | 2090 | 5 | 125 | 0 | 1210 | 0 | 0 | 0 |
|  |  |  |  |  |  |  |  |  |
| 11 | 450 | 155 | 15 | 0 | 100 | 30 | 0 | 0 |
| 11 | 480 | 0 | 260 | 0 | 320 | 0 | 0 | 0 |
| 11 | 575 | 0 | 5 | 0 | 455 | 0 | 0 | 0 |
| 11 | 195 | 0 | 90 | 0 | 780 | 0 | 0 | 0 |
| 11 | 490 | 15 | 5 | 0 | 450 | 160 | 10 | 0 |
| 11 | 155 | 15 | 15 | 0 | 690 | 420 | 0 | 0 |
| 11 | 505 | 245 | 15 | 0 | 350 | 110 | 0 | 0 |
| 11 | 895 | 315 | 95 | 0 | 805 | 25 | 5 | 0 |
| 11 | 705 | 280 | 45 | 0 | 530 | 5 | 0 | 0 |
| 11 | 215 | 0 | 390 | 0 | 770 | 40 | 0 | 0 |
| 11 | 430 | 0 | 0 | 0 | 710 | 10 | 5 | 0 |
| 11 | 1530 | 20 | 60 | 0 | 855 | 70 | 0 | 0 |
| 11 | 400 | 170 | 470 | 0 | 1350 | 265 | 0 | 0 |
| 11 | 1155 | 5 | 255 | 0 | 1090 | 240 | 0 | 0 |
| 11 | 245 | 5 | 65 | 0 | 220 | 40 | 0 | 0 |
| 11 | 185 | 95 | 10 | 0 | 550 | 105 | 0 | 0 |
| 11 | 690 | 0 | 115 | 0 | 365 | 0 | 0 | 0 |
| 11 | 145 | 0 | 0 | 0 | 435 | 570 | 0 | 0 |
| 11 | 665 | 50 | 550 | 0 | 315 | 125 | 30 | 0 |
| 11 | 370 | 15 | 70 | 0 | 1590 | 245 | 0 | 0 |
|  |  |  |  |  |  |  |  |  |
| 12 | 2375 | 25 | 390 | 0 | 2170 | 0 |  |  |
| 12 | 1760 | 0 | 360 | 0 | 2715 | 5 |  |  |
| 12 | 1480 | 10 | 315 | 0 | 650 | 0 |  |  |
| 12 | 1665 | 0 | 140 | 0 | 3265 | 0 |  |  |
| 12 | 1220 | 50 | 135 | 0 | 565 | 0 |  |  |
| 12 | 435 | 0 | 5 | 0 | 1640 | 5 |  |  |
| 12 | 1290 | 10 | 105 | 0 | 1835 | 10 |  |  |
| 12 | 2045 | 0 | 250 | 0 | 1685 | 0 |  |  |
| 12 | 1970 | 5 | 455 | 0 | 2940 | 0 |  |  |
| 12 | 2345 | 0 | 150 | 0 | 680 | 0 |  |  |
| 12 | 600 | 10 | 45 | 0 | 1125 | 0 |  |  |
| 12 | 755 | 65 | 260 | 0 | 1250 | 0 |  |  |
| 12 | 875 | 0 | 450 | 0 | 1280 | 0 |  |  |
| 12 | 720 | 0 | 0 | 0 | 2110 | 0 |  |  |
| 12 | 805 | 0 | 75 | 0 | 865 | 15 |  |  |
| 12 | 1150 | 0 | 130 | 0 | 885 | 0 |  |  |
| 12 | 450 | 0 | 120 | 0 | 850 | 0 |  |  |
| 12 | 2725 | 0 | 25 | 0 | 210 | 0 |  |  |
| 12 | 815 | 0 | 0 | 0 | 1445 | 0 |  |  |
| 12 | 1700 | 0 | 40 | 0 | 1645 | 0 |  |  |
|  |  |  |  |  |  |  |  |  |
| 13 | 1465 | 235 | 255 | 0 | 390 | 45 | 60 | 0 |
| 13 | 725 | 0 | 150 | 0 | 690 | 0 | 45 | 0 |
| 13 | 760 | 5 | 105 | 0 | 1135 | 25 | 5 | 0 |
| 13 | 1070 | 35 | 110 | 0 | 1020 | 0 | 15 | 0 |
| 13 | 1085 | 10 | 55 | 0 | 840 | 10 | 20 | 0 |
| 13 | 1160 | 10 | 155 | 0 | 520 | 25 | 25 | 0 |
| 13 | 390 | 0 | 135 | 0 | 2740 | 40 | 30 | 0 |
| 13 | 1940 | 70 | 35 | 0 | 375 | 0 | 30 | 0 |
| 13 | 1635 | 5 | 175 | 0 | 430 | 0 | 50 | 0 |
| 13 | 295 | 0 | 75 | 0 | 90 | 0 | 30 | 0 |
| 13 | 995 | 0 | 270 | 0 | 465 | 0 | 30 | 0 |
| 13 | 980 | 0 | 5 | 0 | 1105 | 0 | 50 | 0 |
| 13 | 600 | 0 | 175 | 0 | 710 | 70 | 10 | 0 |
| 13 | 910 | 0 | 755 | 0 | 1910 | 245 | 35 | 0 |
| 13 | 1665 | 0 | 685 | 0 | 985 | 60 | 15 | 0 |
| 13 | 650 | 0 | 240 | 0 | 1785 | 0 | 5 | 0 |
| 13 | 740 | 0 | 255 | 0 | 905 | 20 | 20 | 0 |
| 13 | 615 | 0 | 180 | 0 | 2205 | 20 | 40 | 0 |
| 13 |  |  |  |  | 2325 | 0 | 20 | 0 |
| 13 |  |  |  |  | 2195 | 30 | 45 | 0 |
